# Do elevated symptoms of depression predict adherence and outcomes in the UPBEAT randomised controlled trial of a lifestyle intervention for obese pregnant women?

**DOI:** 10.1186/s12884-018-2004-x

**Published:** 2018-09-18

**Authors:** Emma Molyneaux, Shahina Begum, Annette L. Briley, Paul T. Seed, Louise M. Howard, Lucilla Poston, on behalf of the UPBEAT consortium

**Affiliations:** 1 Section of Women’s Mental Health, Institute of Psychiatry, Psychology & Neuroscience at King’s College London, London, England; 2 Department of Women and Children’s Health, King’s College, London, England

**Keywords:** Obesity, Depression, Pregnancy, Lifestyle intervention, Gestational diabetes, Gestational weight gain

## Abstract

**Background:**

Lifestyle interventions for obese pregnant women have been widely researched but little is known about predictors of low adherence or poor outcomes. This study evaluated the prospective associations between elevated symptoms of antenatal depression and gestational diabetes, adherence and gestational weight gain in a large RCT of a behavioural intervention for obese pregnant women. The effect of the intervention on symptoms of depression at follow-up was also examined.

**Methods:**

The UPBEAT RCT randomised 1555 obese pregnant women to receive a dietary and physical activity lifestyle intervention or standard care. Symptoms of antenatal depression were assessed with the Edinburgh Postnatal Depression Scale at baseline (15^+ 0^–18^+ 6^ weeks’ gestation) and follow-up (27^+ 0^–28^+ 6^ weeks’ gestation). Gestational diabetes was assessed with an oral glucose tolerance test at 27^+ 0^–28^+ 6^ weeks’ gestation. Adherence was pre-defined as receiving at least 5 of 8 intervention sessions. Gestational weight gain was calculated as the difference between pre-pregnancy weight (estimated as measured baseline weight minus 1.25 kg) and last measured weight at 34^+ 0^–36^+ 0^ weeks’ gestation. Due to substantial missing data in certain variables, multiple imputation was used to impute missing data. Women who were no longer pregnant at 27^+ 0^–28^+ 6^ weeks’ gestation were excluded from the sample for these analyses.

**Results:**

One thousand five-hundered twenty-six women were included in these analyses following multiple imputation; 797 (52.2%) had complete data. 13.4% had elevated symptoms of antenatal depression at baseline. There was no evidence for associations between antenatal depression status and gestational diabetes (adjusted OR 0.80, 95%CI 0.52 to 1.22, *p* = 0.30), adherence (adjusted OR 1.16, 95%CI 0.63 to 2.15, *p* = 0.63) or gestational weight gain (adjusted regression coefficient 0.52, 95%CI -0.26 to 1.29, *p* = 0.19). The intervention was not associated with change in depressive symptoms at follow-up (regression coefficient 0.003, 95%CI -0.49 to 0.49, *p* = 0.99). Similar results were obtained in complete case analyses.

**Conclusions:**

Elevated symptoms of antenatal depression did not predict gestational diabetes, adherence or gestational weight gain in this large RCT of a lifestyle intervention for obese pregnant women. The intervention also did not influence symptoms of depression at follow-up. Obese pregnant women with elevated symptoms of depression should not be excluded from lifestyle interventions.

**Trial registration:**

ISRCTN89971375. Registered 28 November 2008.

**Electronic supplementary material:**

The online version of this article (10.1186/s12884-018-2004-x) contains supplementary material, which is available to authorized users.

## Background

In recent years, there has been an abundance of research examining lifestyle interventions for obese pregnant women that aim to improve diet, increase physical activity and reduce gestational weight gain. However, these interventions often have limited effectiveness, and low adherence or high drop-out rates are common [1, 2]. There has been little research into predictors of adherence or outcomes in these interventions for pregnant women, but studies of weight management interventions for non-pregnant adults have found that elevated symptoms of depression can be associated with higher attrition and lower adherence [3]. Depression is common during pregnancy and systematic review evidence has demonstrated that obese pregnant women are more likely to experience depression than normal weight women [4]. In addition, antenatal depression has been associated with excessive gestational weight gain in some, although not all, observational studies [5–7]. It is important to identify whether depression is a risk factor for poorer outcomes in lifestyle interventions for obese women during pregnancy.

Amongst the few previous studies examining these associations in pregnancy, one found no evidence that symptoms of depression predicted adherence to the intervention or gestational weight gain in a randomised controlled trial (RCT) of a diet and exercise intervention among 205 obese pregnant women [8]. However, this study was underpowered to examine clinical outcomes of interest such as gestational diabetes, and requires replication. It is also necessary to examine whether taking part in a lifestyle intervention influences symptoms of depression among pregnant women. The few findings on this topic to date have been inconsistent [8–10].

This study examined the prospective associations between elevated symptoms of antenatal depression and 1) gestational diabetes, 2) intervention adherence, and 3) gestational weight gain in the UPBEAT trial, a large RCT of a complex behavioural intervention for obese pregnant women. The effect of the intervention on symptoms of depression at follow-up was also examined.

## Methods

### The UPBEAT trial

The UPBEAT trial (UK Pregnancies Better Eating and Activity Trial) is a multicentre randomised controlled trial of a complex behavioural intervention for obese pregnant women [11]. The intervention focused on improving diet and increasing physical activity with the aim of reducing the risk of gestational diabetes among obese pregnant women, and of delivery of large for gestational age infants [11]. Women randomised to the intervention attended up to eight weekly group sessions delivered by health trainers, and received an intervention handbook, a DVD of pregnancy-specific exercise routines, a pedometer and a log book for recording weekly goals. Women in the control group received standard antenatal care according to the provision in their local area.

For the UPBEAT trial, obese pregnant women (body mass index [BMI] ≥30 kg/m^2^ at their first antenatal appointment) with a singleton pregnancy were recruited between 15^+ 0^ and 18^+ 6^ weeks’ gestation. Exclusion criteria for the trial were lack of informed consent, BMI < 30 kg/m^2^, ≤16 years old, multiple pregnancy, currently being prescribed metformin, and pre-existing medical conditions including diabetes mellitus, hypertension requiring treatment, thyroid disease or current psychosis. In addition, women with known miscarriage, termination, fetal death in utero or preterm birth prior to the first follow-up interview (27^+ 0^ to 28^+ 6^ weeks’ gestation) were excluded from the sample for these analyses.

Women were recruited from eight NHS hospital trusts in the UK between March 2009 and June 2014. Ethical approval for the study was obtained in all centres (UK integrated research application system reference: 09/H0802/5) and the trial was registered with Current Controlled Trials (ISCRTN89971375). Randomisation was performed online using the MedSciNet™ secure data management system, minimised based on age, ethnicity, BMI and centre of recruitment. Given the nature of the intervention, study staff and participants were not blinded to intervention status.

### Measures

#### Antenatal depression

Symptoms of antenatal depression were assessed during the baseline visit (15^+ 0^ to 18^+ 6^ weeks’ gestation) and the first follow-up visit (27^+ 0^ to 28^+ 6^ weeks’ gestation) using the Edinburgh Postnatal Depression Scale (EPDS [12]). A validated cut-off score of ≥13 was used to indicate elevated symptoms of antenatal depression [13]. The difference between EPDS score at baseline and follow-up was calculated to indicate change in depressive symptoms.

#### Gestational diabetes

All women were given an oral glucose tolerance test (75 g load) at 27^+ 0^ to 28^+ 6^ weeks’ gestation and the International Association of Diabetes and Pregnancy Study Groups criteria were used to diagnose gestational diabetes (fasting venous capillary glucose ≥ 5.1 mmol/L and/or 1 h venous glucose ≥10 mmol/L and/or 2 h venous glucose ≥8.5 mmol/L) [14]. All women in the study who met the diagnostic criteria for gestational diabetes were referred for antenatal diabetic services at their local centre.

#### Adherence

High adherence was defined as receiving at least five out of eight intervention sessions (as recommended in the UPBEAT trial protocol [15]) in person, over the phone or via email. Previous analyses in UPBEAT found no evidence that mode of session delivery influenced the primary outcomes [11]. There was also no difference in the proportion of women attending sessions in person, compared with receiving them over the phone or via email, based on depression status (*p* = 0.89).

#### Gestational weight gain

Gestational weight gain (in kg) was calculated as the difference between estimated pre-pregnancy weight and last objective pregnancy weight measured between 34^+ 0^ to 36^+ 0^ weeks’ gestation. As pre-specified in the protocol, estimated pre-pregnancy weight was calculated from weight measured at trial entry with 1.25 kg deducted for first trimester weight gain (1.25 kg is the midpoint of the first trimester weight gain range specified by the Institute of Medicine guidelines [16]).

#### Confounders

Baseline BMI (calculated from measured height and weight at 15^+ 0^ to 18^+ 6^ weeks’ gestation), study centre, socio-demographic factors and parity were included as potential confounders in this study. Details of the assessment and coding of the socio-demographic variables (age, ethnicity, relationship status, highest educational level, household income, and index of multiple deprivation) are given in Table 1. All socio-demographic factors except age were self-reported during the baseline interview.

**Table 1:** Assessment of socio-demographic confounders

Variable	Description
Age	Participants reported their age when they were approached to participate in the study in early pregnancy.
Ethnicity	Participants reported their main ethnicity (European, Indian, Pakistani, Bangladeshi, Afro Caribbean, African, Middle Eastern, Far East Asian, South East Asian or Other) which was classified as White, Black, Asian or Other.
Relationship status	Relationship status was indicated by whether or not participants were currently living with their partner. This was used instead of marital status, which provided limited information on relationship status in this sample as the majority of women reported having never been married.
Highest educational level	Highest educational level was self-reported as none, GCSE or equivalent vocational qualification, A level or equivalent, first degree or higher degree. This was coded for analyses as none/GCSE, A level/vocational or degree level. GCSEs are public exams usually taken in school at 15–16 years; A levels are public exams usually taken in school at 17–18 years, and are generally required for University entrance.
Household income	Household income (participant and partner, pre-tax per year) was recorded as <£12,688, £12,688–£17,628, £17,629–£23,452, £23,453–£32,500 or > £32,500.
Index of multiple deprivation	Index of multiple deprivation [27] is assessed at the lower layer super output area (LSOA) level. Women were given an index of multiple deprivation score based on their postcode. Based on data for all LSOAs in England and Scotland, each LSOA was defined based on its quintile group [28].
Parity	Parity was self-reported and defined as the number of previous pregnancies going beyond 20 weeks’ gestation.

### Main analyses

All analyses were conducted in Stata 14 (StataCorp, 2015, College Station, Texas). There was no missing data for the majority of baseline variables; however income and EPDS score were missing for 15.5% and 12.6% of participants respectively. There was also missing data in the outcome variables, with approximately 15% missing for GDM and over 20% missing for EPDS at follow-up and gestational weight gain (see Additional file 1). Missing data reduces statistical power in complete case analysis and can lead to bias if participants with missing data differ from those with complete data. Based on the assumption of missing at random, which was plausible in this dataset (see Additional files 1, 2 and 3), multiple imputation by chained equations was used to impute missing data [17]. All analysis variables were included in the imputation model alongside other relevant auxiliary variables to improve the prediction of missing values and increase the plausibility of missing at random [18]. The number of imputed datasets was based on the proportion of missing data as simulation studies have suggested that the number of imputed datasets should be greater than the proportion of participants with missing data [19]. Sixty imputed datasets were produced. Statistical analyses were then carried on each imputed dataset separately and combined using Rubin’s rules [20]. Full details of the imputation are provided in the Additional files 1, 2 and 3.

Using the imputed data, characteristics of the study sample were summarised for socio-demographic variables, BMI, parity and centre of recruitment. Characteristics of women with EPDS scores ≥13 and EPDS scores < 13 were presented as frequencies or means (with standard deviations (SD)) and compared using univariate regression analyses. The relationship between antenatal depression at 15^+ 0^ to 18^+ 6^ weeks’ gestation (independent variable) and gestational diabetes (dependent variable) was then examined using univariate logistic regression. The interaction of depression and intervention group on risk of gestational diabetes was tested and if this was found to be significant, subgroup analyses were performed to examine the intervention and control groups separately. If the interaction effect was not significant, the association between antenatal depression and gestational diabetes was examined in the sample as a whole. The relationship between EPDS score (as a continuous variable) and gestational diabetes was also examined using unadjusted logistic regression. In addition, fasting blood glucose levels (mmol/L) and gestation at the time of the oral glucose tolerance test were compared between women with and without elevated symptoms of antenatal depression (EPDS < 13 and EPDS ≥13). Multiple logistic regression was then used to examine the association between depression (EPDS≥13) and gestational diabetes adjusted for potential confounders (BMI, age, ethnicity, relationship status, educational level, household income, index of multiple deprivation, parity and study centre). The association between antenatal depression and high adherence (receiving five or more intervention sessions out of a possible total of eight) was then examined for women in the intervention group only, using logistic regression with and without adjusting for confounders (as above). The relationship between EPDS score, as a continuous variable, and adherence was also examined. Next, the association between antenatal depression and gestational weight gain was examined using linear regression, again with adjustment for confounders. Assumptions of linear regression were checked (normality of residuals, homoscedasticity and independence of predictors) and one outlier for gestational weight gain was removed prior to imputation. The relationship between EPDS score, as a continuous variable, and gestational weight gain was also examined. Finally, the effect of the intervention on the change in symptoms of depression from baseline to follow-up was examined using linear regression. The main unadjusted and adjusted analyses were then repeated using complete case analysis, for comparison with the imputed data.

## Results

**Fig. 1:**
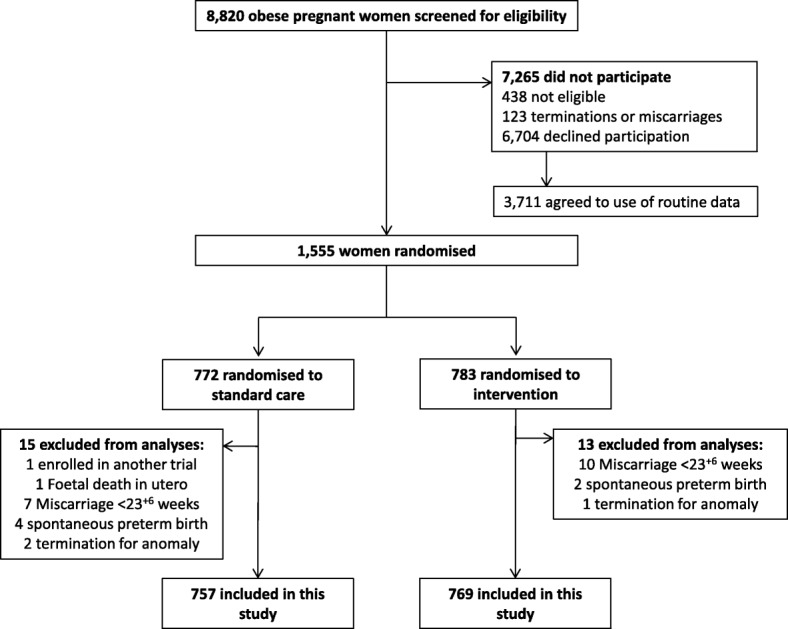
Participant flow diagram

In total, 1555 women were recruited into the UPBEAT trial. One participant was excluded after enrolment in another trial, leaving 1554 participants. The CONSORT diagram is given in Fig. 1. The number of participants included in the trial represented 19% of the 8259 eligible women approached to participate (8820 women with BMI ≥30 kg/m^2^ were approached, of whom 561 were not eligible to participate). Women with known miscarriage, termination, fetal death in utero or preterm birth prior to 27^+ 0^ to 28^+ 6^ weeks’ gestation (*n* = 28) were also excluded from the sample for these analyses. One thousand five hundered twenty-six women were therefore included in this study (757 randomised to standard antenatal care and 769 randomised to the intervention).

Among the 1526 women included in this study, 797 (52.2%) had complete data for all analysis variables. In total, 47.87% (*n* = 729) of participants had missing data for at least one analysis variable but only 5.0% (*n* = 76) had missing data for more than three analysis variables. Overall, 6.2% of the total observations in the analysis variables were missing. 12.6% of women were missing EPDS score at baseline but there was complete data for most other baseline characteristics except for household income, which was missing for 15.5% of women (see Additional file 1). The pattern of missing data was examined and the assumption of missing at random was plausible (see Additional file 2). Multiple imputation by chained equations [17] was therefore performed (see Additional file 3) with 60 imputed datasets produced. Overall, characteristics of the imputed data were similar to the observed data (see Additional file 3). For example, 13.1% of women scored ≥13 on the EPDS at baseline in the observed data, and 13.4% in the imputed data. The mean length of follow-up was 10.9 weeks (SD 1.6) both for women with EPDS scores < 13 at baseline and for women with EPDS scores ≥13.

### Sample characteristics

Characteristics of the sample (based on the imputed data) are given in Table 2. Approximately half of the sample was recruited from hospital trusts in London (49.3%). The mean age of the sample was 30.5 years (±5.5) and 43.3% of women were nulliparous. The majority of women (63.0%) were of white ethnicity, and 25.5% of black ethnicity. Just over three quarters of participants (77.1%) were living with a partner. One fifth (20.5%) of participants had no qualifications beyond GCSEs or equivalent. A quarter of participants (24.6%) reported a household income of £12,688 or less per year, whilst 37.8% reported a household income greater than £32,500 per year. The majority of participants (77.8%) lived in areas in the highest or second highest quintiles of deprivation.

**Table 2:** Characteristics of the sample and associations with antenatal depression status

		Total sample %	Antenatal depression status		
			EPDS < 13	EPDS ≥ 13	*p* value
Total; %		–	86.6	13.4	–
Centre; %	Guys’ and St Thomas’^1^	24.4	84.2	15.8	0.21
	King’s College Hospital^1^	17.8	84.5	15.5	
	Newcastle	15.7	89.9	10.1	
	Glasgow	17.0	89.1	10.9	
	Manchester	9.1	88.5	11.6	
	Bradford	3.3	82.4	17.7	
	Sunderland	5.4	91.6	8.4	
	St Georges’^1^	7.1	83.4	16.6	
Parity; %	0	43.3	87.0	13.0	0.17
	1	33.5	88.5	11.5	
	2	15.3	82.4	17.6	
	3+	7.9	85.1	14.9	
Age; mean (sd)		30.5 (5.5)	30.5 (5.4)	30.1 (5.7)	0.33
Main ethnicity; %	White	63.0	89.4	10.6	< 0.01
	Black	25.5	82.3	17.7	
	Asian	6.0	80.7	19.3	
	Other	5.5	81.9	18.1	
Relationship status; %	Not cohabiting	22.9	79.8	20.2	< 0.01
	Cohabiting	77.1	88.7	11.3	
Highest educational level^2^; %	None/GCSE	20.5	83.8	16.2	0.02
	A level/Vocational	39.7	85.6	14.4	
	Degree	39.8	89.1	10.9	
Household income; %	<£12,688	24.6	78.5	21.5	< 0.01
	£12,688–17,628	13.5	83.2	16.8	
	£17,629-23,452	10.1	87.2	12.9	
	£23,453-32,500	14.1	90.4	9.7	
	>£32,500	37.8	91.7	8.3	
Index of multiple deprivation quintiles; %	1 (least)	4.1	94.0	6.0	0.03
	2	6.7	85.1	15.0	
	3	11.4	88.6	11.4	
	4	34.3	88.9	11.2	
	5 (most)	43.5	84.0	16.0	
BMI (kg/m^2^); %	30–35	49.7	86.7	13.3	0.92
	35–40	32.1	86.3	13.7	
	≥40	18.2	87.1	12.9	

Sample characteristics based on imputed data. ^1^London. ^2^GCSEs: public exams usually taken in school at 15–16 years; A levels: public exams usually taken in school at 17–18 years

At 15^+ 0^ to 18^+ 6^ weeks’ gestation, the median EPDS score was 6 (IQR 3–10). Elevated symptoms of antenatal depression (≥13 on the EPDS) were observed in 13.4% of the sample. The proportion of women with elevated symptoms of antenatal depression did not differ between the intervention groups (control group: 13.2%, intervention group 13.5%; *p* = 0.90). However, the prevalence of antenatal depression was associated with the majority of other sample characteristics (see Table 2). Elevated symptoms of depression were least common among white women (10.6%) and more common among all other ethnic groups (17.7–19.3%). The prevalence was also higher among women who were not cohabiting (20.2 vs. 11.3%). In addition, the prevalence of elevated symptoms of antenatal depression differed by all indicators of socio-economic status. For example, 21.5% for women with household income less than £12,688 scored ≥13 on the EPDS, compared with 8.3% for women with household income over £32,500. Finally, elevated symptoms of antenatal depression were reported most often in Bradford (17.7%) followed by the three London study sites (15.5–16.6%) and least often in Sunderland (8.4%) and Newcastle (10.1%).

### Gestational diabetes

The overall prevalence of gestational diabetes, based on imputed data, was 28.2%. 26.3% of women with elevated symptoms of antenatal depression developed gestational diabetes compared with 28.5% of women without elevated symptoms of depression. Fasting blood glucose levels did not differ between women with and without elevated symptoms of depression (EPDS ≥13: mean 4.6 mmol/L (SD 0.6); EPDS< 13: mean 4.7 mmol/L (SD 0.6); *p* = 0.25), nor did gestational age at the time of the oral glucose tolerance test (EPDS ≥13: mean 27^+ 6^ weeks; EPDS < 13: mean: 28^+ 0^ weeks; *p* = 0.26), based on observed data.

Using unadjusted logistic regression, there was no evidence of an association between elevated symptoms of antenatal depression and gestational diabetes (OR 0.89, 95%CI 0.58–1.32, *p* = 0.56), no evidence for an interaction of depression status and intervention group on risk of gestational diabetes (*p* = 0.41), and no evidence of an association between EPDS score (as a continuous predictor) and gestational diabetes (OR 0.99, 95%CI 0.96–1.01, *p* = 0.37). Finally, there was also no evidence for an association between elevated symptoms of antenatal depression and gestational diabetes after adjusting for confounders (OR 0.80, 95%CI 0.52–1.22, *p* = 0.30).

### Adherence

Among women in the intervention group, 81.1% received at least five of the eight intervention sessions (the pre-defined threshold for adherence). Examining adherence by antenatal depression status, 80.0% of women with elevated symptoms of depression were adherent compared with 81.3% of women without elevated symptoms. Elevated antenatal depression symptoms were not associated with high adherence before or after adjusting for confounders (unadjusted OR 0.92, 95%CI 0.54–1.57, *p* = 0.76; adjusted OR 1.16, 95%CI 0.63–2.15, *p* = 0.63). There was also no evidence of an association between EPDS score (as a continuous predictor) and adherence (OR 1.00, 95%CI 0.96–1.04, *p* = 0.99).

### Gestational weight gain

Mean gestational weight gain was 7.41 kg. Based on antenatal depression status, mean gestational weight was 7.58 kg among women with elevated symptoms of depression, compared with 7.39 kg among women without elevated symptoms. There was no evidence for a difference in the total period of time over which gestational weight gain was assessed, based on baseline antenatal depression status (EPDS < 13: 18.1 weeks (SD 3.3); EPDS ≥13: 18.5 weeks (SD 5.9), *p* = 0.23).

There was no association between antenatal depression status and gestational weight gain in the unadjusted regression analysis (regression coefficient 0.19, 95%CI -0.59 to 0.97, *p* = 0.63), no evidence for an interaction of depression and intervention group on gestational weight gain (*p* = 0.41), and no evidence of an association between EPDS score (as a continuous predictor) and gestational weight gain (regression coefficient 0.04, 95%CI -0.02 to 0.09, *p* = 0.22). There was also no significant association between elevated symptoms of antenatal depression and higher gestational weight gain after adjusting for confounders (adjusted regression coefficient 0.52, 95%CI -0.26 to 1.29, *p* = 0.19).

### Change in depressive symptoms

At follow-up, the median EPDS score was 5 (IQR 2–9). 11.1% of women in the intervention group scored ≥13 on the EPDS at follow-up compared with 11.6% in the control group. The mean reduction in EPDS score from baseline to follow-up was 0.77 points in both the intervention and control groups. There was no association between intervention group and change in EPDS symptoms (regression coefficient 0.003, 95%CI -0.49 to 0.49, *p* = 0.99).

### Complete case analysis

For the complete case analyses, sample size varied based on the amount of missing data in the included variables. There was no evidence for an association between elevated symptoms of antenatal depression and gestational diabetes in the unadjusted (OR 0.72, 95%CI 0.47–1.12, *p* = 0.14; *n* = 1143) or adjusted analyses (OR 0.70, 95%CI 0.42–1.17, *p* = 0.17; *n* = 972), and no evidence for an interaction of depression status and study group on gestational diabetes (*p* = 0.29). There was also no evidence for an association between elevated antenatal depression symptoms and high adherence in the unadjusted (OR 0.84, 95%CI 0.49–1.43 *p* = 0.51; *n* = 679) or adjusted (OR 0.99, 95%CI 0.49–2.00, *p* = 0.98; *n* = 567) analyses. Elevated symptoms of antenatal depression and gestational weight gain were not associated in the unadjusted analysis (regression coefficient 0.62, 95%CI -0.26 to 1.50, *p* = 0.17; *n* = 948) but there was a significant association after adjusting for confounders (regression coefficient 1.11, 95%CI 0. Fifteen to 2.07, *p* = 0.02; *n* = 805). There was no evidence for an interaction between antenatal depression status and intervention group on total gestational weight gain (*p* = 0.75). Finally, there was no evidence for an association between intervention group and change in severity of symptoms of depression at follow-up (regression coefficient − 0.10, 95%CI -0.58 to 0.38, *p* = 0.68, *n* = 1140).

## Discussion

Elevated symptoms of antenatal depression (EPDS score ≥ 13) were reported by almost one in seven (13.4%) obese pregnant women participating in the UPBEAT trial. However, elevated depressive symptoms did not predict risk of gestational diabetes, adherence or gestational weight gain. There was also no evidence that receiving the dietary and physical activity lifestyle intervention during pregnancy influenced symptoms of depression at follow-up.

Our findings are in keeping with a previous RCT of a lifestyle intervention for obese pregnant women which found no association between symptoms of antenatal depression and intervention adherence or gestational weight gain (Bogaerts et al. [8]). In this study, we extended previous research by examining gestational diabetes, which many previous trials have been underpowered to assess, but again found no association with antenatal depression. We also found no interactions between depression status and intervention group, meaning that the effectiveness of the intervention for the outcomes examined did not differ between women with and without antenatal depression. Future studies could examine whether antenatal depression is associated with differences in diet or physical activity in lifestyle interventions for obese pregnant women.

The intervention in the UPBEAT RCT was found to have no effect on symptoms of antenatal depression at follow-up. Previous findings on this topic are mixed. One small study from Spain, conducted by Perales et al. (2015), found that overweight and obese pregnant women randomised to an exercise programme had a lower prevalence of depression in late pregnancy than women in the control group [10]. Bogaerts et al., in contrast, found that their intervention had no effect on symptoms of antenatal depression, but symptoms of anxiety were reduced compared with controls [8]. Anxiety symptoms were not assessed in the UPBEAT study so this could not been examined here. However, in an large RCT of a lifestyle advice intervention for overweight and obese pregnant women (*n* = 2212), Dodd et al. [9] also found no effect of the intervention on symptoms of depression or anxiety at follow-up, including follow-up at 4 months postpartum. Considering our results in the context of these previous findings it appears that lifestyle interventions during pregnancy, including the extra contact received and any lifestyle change occuring as a result of these interventions, do not reduce symptoms of depression for the majority of obese women. It may be that structured exercise interventions, as used by Perales et al., have more effect on mental health than lifestyle interventions providing counselling around diet and exercise. However more high quality RCTs of structured exercise interventions for pregnant women are needed to conclude whether these are effective and feasible in routine clinical practice. It is important to ensure that mental health services are available for all women who require treatment for depression during pregnancy. No data were available on mental health interventions received by women in the UPBEAT trial, which is a limitation of these analyses.

The UPBEAT RCT included over 1500 obese pregnant women. This large sample size is a key strength of the study, and enabled clinical outcomes such as gestational diabetes to be assessed. The main findings of the UPBEAT trial (reported elsewhere [11]) showed that the intervention was not effective in preventing gestational diabetes (the primary maternal outcome), although it did improve diet quality and physical activity, reduce gestational weight gain and reduce maternal adiposity. The assessment of gestational diabetes was a particular strength of this study; the International Diabetes and Pregnancy Study Groups criteria [14] were used to diagnose gestational diabetes and all women were given a glucose tolerance test at 27^+ 0^ to 28^+ 6^ weeks’ gestation rather than relying on clinical records to obtain outcome data.

A number of limitations of this study must also be taken into account. Only 19% of the 8259 eligible women approached about the UPBEAT RCT agreed to participate. This is similar to recruitment rates in other studies of lifestyle interventions for overweight or obese pregnant women [21–23] but limits the representativeness of the sample and the likely generalisability of the findings. Routine data (collected from non-participants who gave permission) showed that women who did not participate were younger, more likely to be of black ethnicity and had slightly lower BMI. Routine data from non-participants could not be collected on depression status but women with depression might be expected to be less likely to participate. The prevalence of elevated symptoms of antenatal depression observed in the UPBEAT sample at baseline (approximately 13% of women scored ≥13 on the EPDS) is similar to other prevalence estimates during pregnancy [24]. However, the UPBEAT study included obese and largely low-income women, and both of these characteristics have been associated with increased risk of antenatal depression [25, 26], which may suggest that women with depression were underrepresented in the sample. It is also important to note that the EPDS is a screening measure which identifies women with elevated symptoms of depression rather than those meeting diagnostic criteria, meaning that conclusions cannot be drawn about women with depressive disorders from this study. However, the EPDS is widely used to assess antenatal depression in research and clinical practice and has been validated to indicate probable depression during pregnancy [13]. The main analyses were conducted using the EPDS as a dichotomous variable, but additional analyses using the EPDS as a continuous predictor gave similar findings.

Finally, missing data is a common problem in longitudinal studies including the UPBEAT RCT, leading to loss of statistical power and potential bias. This study included 1526 women, of whom only 52% (*n* = 797) had complete data for all analysis variables. However, the amount of missing data for each participant was generally low, with 95% of participants missing three or fewer variables. The main analyses for this paper were conducted using multiple imputation to impute missing data, which gives unbiased estimates based on the assumption that data are missing at random (i.e. that missing values can be predicted as a function of the observed data). It is not possible to conclusively test whether data are missing at random, but this was highly plausible in this sample. There were rich baseline data available on sample characteristics, the majority of which had no missing data, and these characteristics predicted missing data in the follow-up interviews. It is often argued that data on mental health is unlikely to be missing at random as participants with poor mental health may be more likely to have missing data [18]. However, in this study the vast majority of missing baseline EPDS data occurred during a short period of the study in which this variable was not recorded, so this missing data is not related to unmeasured participant characteristics and can be characterised as missing at random.

Our conclusions are also strengthened by a complete case analysis which gave very similar findings. The only difference observed between the complete case and imputed analyses is that elevated symptoms of antenatal depression were significantly associated with increased gestational weight gain after adjustment for confounders in the complete case analysis, which was not the case in the imputed analyses. However, it is plausible that this association in the complete case analyses was the result of bias from the exclusion of participants with missing data. A previous study on this topic also found no association between symptoms of antenatal depression and gestational weight gain [8], in keeping with the results of the imputed analyses.

## Conclusions

Elevated symptoms of depression are common among obese pregnant women. In the UPBEAT RCT there was no association between elevated symptoms of antenatal depression at baseline and adherence to the intervention, risk of gestational diabetes, or gestational weight gain among obese pregnant women. There was also no evidence that the effectiveness of the intervention differed based on antenatal depression status, or that the intervention influenced symptoms of depression at follow-up. We conclude that obese pregnant women with elevated symptoms of depression should not be excluded from lifestyle interventions due to concerns about intervention adherence and effectiveness, or concerns about impact on depressive symptomatology. Awareness of depression among health professionals providing care for obese pregnant women nonetheless remains crucial for detection and treatment of this disorder.

## Additional files

Additional file 1: Missing data for participants included in these analyses (*n* = 1526). The data in this section describe the proportion of missing data for each analysis variable. (PDF 68 kb)

Additional file 2: Mechanisms of missing data. The data in this section describe the patterns of missing data and examine the association of participant characteristics with missing data. (PDF 97 kb)

Additional file 3: Multiple imputation by chained equations. This section provides detailed information on the methods of the multiple imputation, and presents data comparing the characteristics of observed and imputed data. (PDF 151 kb)

## Abbreviations

95% CI: 95% confidence interval
BMI: Body mass index
EPDS: Edinburgh Postnatal Depression Scale
OR: Odds ratios
RCT: Randomised controlled trial
SD: Standard deviation
UPBEAT: UK Pregnancies Better Eating and Activity Trial
