# Supplementary material for: Do elevated symptoms of depression predict adherence and outcomes in the UPBEAT randomised controlled trial of a lifestyle intervention for obese pregnant women?
Source: BMC Pregnancy Childbirth. 2018 Sep 18;18:378. doi: 10.1186/s12884-018-2004-x (PMC6142329; doi:10.1186/s12884-018-2004-x)
Supplement: Supplementary file 1 — Missing data for participants included in these analyses (n = 1526). The data in this section describe the proportion of missing data for each analysis variable. (PDF 68 kb) [file 12884_2018_2004_MOESM1_ESM.pdf]

Additional file 1: Missing data for participants included in these analyses (n=1,526)

In total, 1,555 women were recruited into the UPBEAT trial. One participant was excluded after enrolment in another trial and women with known miscarriage, termination, fetal death in utero or preterm birth prior to 27^{+0} to 28^{+6} weeks' gestation (n=28) were also excluded from the sample for these analyses. 1,526 women were therefore included in this study.

Among the 1,526 women included in this study, 47.8% (n=729) had missing data on at least one analysis variable but only 5.0% (n=76) had missing data for more than three analysis variables. Overall, 6.2% of the total observations in the analysis variables were missing.

Table 1: Observed and missing data for each analysis variable

Variable	Observed; n (%)*	Missing; n (%)
Baseline EPDS score	1,334 (87.4)	192 (12.6)
Study centre	1,526 (100)	0
BMI	1,526 (100)	0
Parity	1,526 (100)	0
Age	1,526 (100)	0
Ethnicity	1,526 (100)	0
Living with partner	1,526 (100)	0
Household income per year	1,290 (84.5)	236 (15.5)
Index of multiple deprivation	1,520 (99.6)	6 (0.4)
Highest educational level	1,526 (100)	0
Randomisation group	1,526 (100)	0
GDM	1,305 (85.5)	221 (14.5)
Number of intervention sessions received	769 (100)*	0
Gestational weight gain	1,093 (71.6)	433 (28.4)
EPDS at follow-up	1,184 (77.6)	342 (22.4)

*Total N=1,526 for all variables except 'number of intervention sessions' received (only available for women in the intervention group; n=769)
