# Supplementary material for: Do elevated symptoms of depression predict adherence and outcomes in the UPBEAT randomised controlled trial of a lifestyle intervention for obese pregnant women?
Source: BMC Pregnancy Childbirth. 2018 Sep 18;18:378. doi: 10.1186/s12884-018-2004-x (PMC6142329; doi:10.1186/s12884-018-2004-x)
Supplement: Supplementary file 2 — Mechanisms of missing data. The data in this section describe the patterns of missing data and examine the association of participant characteristics with missing data. (PDF 97 kb) [file 12884_2018_2004_MOESM2_ESM.pdf]

Additional file 2: Mechanisms of missing data

Mechanisms of missing data ("Missing Completely At Random (MCAR)", "Missing At Random (MAR)" or "Missing Not At Random (MNAR)" [1]) were examined. A number of participant characteristics were associated with missing data (see Table 2), so the data were not missing completely at random. Please note, totals in Table 2 vary due to missing data and high adherence only available for those in the intervention group (n=783)

Data are described as missing at random if the probability of data being missing is a function of observed data. Patterns of missing data were therefore examined to assess the plausibility of missing at random. For example, the majority of missing data for the EPDS at baseline occurred during a particular time period (May 2011 to December 2011), meaning that EPDS missingness can be predicted by time of recruitment and is unlikely to be related to unmeasured participant characteristics. In addition, the majority of missing data at follow-up occurred due to non-attendance or loss to follow-up, which can be predicted by baseline variables including age, ethnicity, centre of recruitment and household income. It is therefore also plausible that missing data for these variables would meet the assumption of missing at random. The richness of the data collection in the UPBEAT sample also contributed substantially to the plausibility of missing at random and the validity of multiple imputation. For example, although there was substantial missing data for income, there was available data on employment status, highest educational level and index of multiple deprivation which were observed for almost all participants and could be used to predict income. The assumption of missing at random was therefore found to be plausible.

Table 2: Associations between participant characteristics and missing data in the overall UPBEAT sample (n=1,554)

		No missing	Some missing	p value
**Centre; n(%)**	Guy's and St Thomas'	194 (50.5)	190 (49.5)	<0.01
	King's College Hospital	115 (41.7)	161 (58.3)	
	Newcastle	124 (51.2)	118 (48.8)	
	Glasgow	158 (59.6)	107 (40.4)	
	Manchester	76 (54.7)	63 (45.3)	
	Bradford	18 (34.0)	35 (66.0)	
	Sunderland	56 (66.7)	28 (33.3)	
	St Georges'	55 (49.6)	56 (50.5)	
**Parity; n(%)**	0	378 (56.1)	296 (43.9)	<0.01
	1	262 (50.8)	254 (49.2)	
	2	106 (44.4)	133 (55.7)	
	3+	50 (40.0)	75 (60.0)	
**Age (years); n(%)**	<20	10 (31.2)	22 (68.8)	<0.01
	20-24	90 (43.1)	119 (56.9)	
	25-29	204 (49.3)	210 (50.7)	
	30-34	263 (53.6)	228 (46.4)	
	≥35	229 (56.1)	179 (43.9)	
**BMI; n(%)**	30-35	387 (50.6)	378 (49.4)	0.71
	35-40	268 (52.8)	240 (47.2)	
	>40	141 (50.2)	140 (49.8)	
**Main ethnicity; n(%)**	White	555 (57.0)	418 (43.0)	<0.01
	Black	163 (40.7)	238 (59.4)	
	Asian	36 (37.9)	59 (62.1)	
	Other	42 (49.4)	43 (50.6)	
**Relationship status; n(%)**	Not cohabiting	150 (41.9)	208 (58.1)	<0.01
	Cohabiting	646 (54.0)	550 (46.0)	
**Highest educational level; n(%)**	None/GCE	136 (42.9)	181 (57.1)	<0.01
	A level/Vocational	295 (47.5)	326 (52.5)	
	Degree	365 (59.3)	251 (40.8)	
**Household income; n(%)**	<£12,688	131 (45.8)	155 (54.2)	<0.01
	£12,688-17628	105 (61.8)	65 (38.2)	
	£17,629-23,452	74 (56.5)	57 (43.5)	
	£23,453-32,500	119 (62.6)	71 (37.4)	
	>£32,500	367 (68.7)	167 (31.3)	
**Index of multiple deprivation quintile; n(%)**	1 (least deprived)	36 (55.4)	29 (44.6)	0.18
	2	60 (58.3)	43 (41.8)	
	3	95 (53.7)	82 (46.3)	
	4	283 (53.1)	250 (46.9)	
	5 (most deprived)	322 (48.1)	348 (51.9)	
**Randomisation group; n(%)**	Intervention	382 (48.8)	401 (51.2)	0.05
	Control	414 (53.7)	357 (46.3)	
**EPDS at baseline; n(%)**	<13	706 (59.8)	475 (40.2)	0.02
	≥13	90 (50.6)	88 (49.4)	
**EPDS at follow-up; n(%)**	<13	720 (68.1)	337 (31.9)	0.06
	≥13	76 (59.8)	51 (40.2)	
**Gestational diabetes; n(%)**	No	596 (61.6)	371 (38.4)	0.42
	Yes	200 (59.2)	138 (40.8)	
**High adherence; n(%)**	No	30 (19.0)	128 (81.1)	<0.01
	Yes	352 (56.3)	273 (43.7)	
**Gestational weight gain; mean(sd)**		7.70 (4.44)	6.87 (4.44)	0.06
