# Supplementary material for: Do elevated symptoms of depression predict adherence and outcomes in the UPBEAT randomised controlled trial of a lifestyle intervention for obese pregnant women?
Source: BMC Pregnancy Childbirth. 2018 Sep 18;18:378. doi: 10.1186/s12884-018-2004-x (PMC6142329; doi:10.1186/s12884-018-2004-x)
Supplement: Supplementary file 3 — Multiple imputation by chained equations. This section provides detailed information on the methods of the multiple imputation, and presents data comparing the characteristics of observed and imputed data. (PDF 151 kb) [file 12884_2018_2004_MOESM3_ESM.pdf]

Additional file 3: Multiple imputation by chained equations

As the assumption of missing at random was plausible, missing data were imputed using multiple imputation by chained equations [2].

All analysis variables were included in the imputation model alongside other relevant auxiliary variables to improve the prediction of missing values and increase the plausibility of missing at random.[3] The number of imputed datasets was based on the proportion of missing data as simulation studies have suggested that the number of imputed datasets should be greater than the proportion of participants with missing data.[4]

Prior to imputation, summary data and plots for each continuous variable were examined for outliers and regression assumptions were checked. One outlier was removed for gestational weight gain (weight gain of 37.25kg, with all other values between -7.65kg and 22.55kg). Linear regression was used to impute normally distributed continuous variables, logistic regression was used to impute dichotomous variables and ordered logistic regression was used to impute ordered categorical variables. Predictive mean matching was used to impute semi-continuous variables (such as EPDS score) and continuous non-normally distributed variables. Intervention group and the interaction of depression and intervention group were also included in the model, with the interaction term passively imputed from EPDS score at baseline and intervention group. A full list of variables included in the imputation model and method of imputation (for variables with missing data) is given in Table 3. Multiple imputation was performed in Stata using the user-written ‘ice’ command [5] then imported to the ‘mi’ format. 60 imputed datasets were produced, each based on 10 cycles of updates for missing values.

After data were imputed, the characteristics of the imputed data were compared with the observed data (for the whole sample of 1,554 women), see Table 4. Characteristics were generally highly comparable between observed and imputed data, for example, 13.1% of women scored ≥13 on the EPDS at baseline in the observed data, and 13.3% in the imputed data. Some differences were observed, for example the prevalence of gestational diabetes was higher in the imputed and observed data. However, this is likely to be explained by the fact that characteristics associated with increased risk of gestational diabetes were also associated with having missing follow-up data. Please note that in Table 4, the imputed data column is marked as not applicable for variables with no missing data as these were not imputed.

Table 3: Variables included in the imputation model

Analysis variables	Method of imputation
Baseline EPDS score	Predictive mean matching
GDM	logit
Number of intervention sessions received	*NA*
Gestational weight gain at the end of pregnancy (pmm)	Predictive mean matching
Study centre	*NA*
BMI	*NA*
Parity	*NA*
Age	*NA*
Ethnicity	*NA*
Living with partner	*NA*
Household income per year (ologit)⁺	ologit
Index of multiple deprivation (ologit)	ologit
Highest educational level	*NA*
Randomisation group	*NA*
EPDS at follow-up (pmm)	pmm
EPDS x randomisation group interaction	Passively imputed
**Auxiliary variables**	**Method of imputation**
GDM in the index pregnancy from records	logit
Smoking	*NA*
Children in household	*NA*
Baseline EQ-5D Anxiety and depression	ologit
Follow-up EQ-5D Anxiety and depression	ologit
Years of education	*NA*
Marital status	*NA*
Preeclampsia	logit
Large for gestational age delivery	logit
Study enrolment status at first follow-up interview	mlogit
Date of recruitment	Predictive mean matching
Employment status	*NA*
Gestational weight gain at first follow-up	Predictive mean matching

Table 4: Characteristics of the overall UPBEAT sample (n=1,554) before and after multiple imputation

		Observed data	Imputed data
**Centre; %**	Guys' and St Thomas'	24.7	*NA*
	King's College Hospital	17.8	*NA*
	Newcastle	15.6	*NA*
	Glasgow	17.1	*NA*
	Manchester	8.9	*NA*
	Bradford	3.4	*NA*
	Sunderland	5.4	*NA*
	St Georges'	7.1	*NA*
**Parity; %**	0	43.4	*NA*
	1	33.2	*NA*
	2	15.4	*NAs*
	3+	8.0	*NA*
**Age; mean (sd)**		30.5 (5.49)	*NA*
**BMI; %**	30-35	49.2	*NA*
	35-40	32.7	*NA*
	≥40	18.1	*NA*
**Main ethnicity; %**	White	62.6	*NA*
	Black	6.1	*NA*
	Asian	25.8	*NA*
	Other	5.5	*NA*
**Relationship status; %**	Not cohabiting	23.0	*NA*
	Cohabiting	77.0	*NA*
**Education; %**	None/GCSE	20.4	*NA*
	A level/Vocational	40.0	*NA*
	Degree	39.6	*NA*
**Household income; %**	<£12,688	21.8	24.7
	£12,688-17628	13.0	13.6
	£17,629-23,452	10.0	10.1
	£23,453-32,500	14.5	14.1
	>£32,500	40.7	37.5
**Index of multiple deprivation quintile; %**	1 (least)	4.2	4.2
	2	6.7	6.7
	3	11.4	11.4
	4	34.4	34.4
	5 (most)	43.3	43.3
**EPDS score at baseline; %**	<13	86.9	86.7
	≥13	13.1	13.3
**EPDS score at follow-up; %**	<13	89.3	88.7
	≥13	10.7	11.3
**GDM; %**	No	74.1	71.4
	Yes	25.9	28.6
**Adherence; %**	Low	20.2	*NA*
	High	79.8	*NA*
**Gestational weight gain (kg); mean (sd)**		7.48 (4.46)	7.41 (4.52)

References

1. Rubin DB. Multiple imputation for non-response in surveys. New York: John Wiley & Sons; 1987.
2. Van Buuren S, Oudshoorn K. Flexible multivariate imputation by MICE. In.: Leiden: TNO Prevention and Health (TNO Publication No. PG/VGZ/99.054); 1999.
3. Sterne JA, White IR, Carlin JB, Spratt M, Royston P, Kenward MG et al. Multiple imputation for missing data in epidemiological and clinical research: potential and pitfalls. BMJ 2009, 338: b2393.
4. Bodner TE. What improves with increased missing data imputations? Structural Equation Modeling: A Multidisciplinary Journal 2008, 15(4): 651-675.
5. Royston P. Multiple imputation of missing values: further update of ice, with an emphasis on interval censoring. Stata Journal 2007, 7(4): 445-464.
